# Tuberculous ventriculitis as a devastating neurological manifestation of immune reconstitution inflammatory syndrome: A case report in immunocompetent patient

**DOI:** 10.1016/j.ijscr.2024.110493

**Published:** 2024-10-19

**Authors:** Andre Marolop Pangihutan Siahaan, Bahagia Willibrordus Maria Nainggolan, Ahmad Brata Rosa, Marsal Risfandi, Andika Pradana, David M.R. Silalahi

**Affiliations:** a Department of Neurosurgery, Faculty of Medicine, Universitas Sumatera Utara, Medan, Indonesia; b Medical Doctor, Universitas Sumatera Utara, Medan, Indonesia; c Faculty of Sport Sciences, Universitas Negeri Medan, Medan, Indonesia; d Department of Pulmonology and Respiratory Medicine, Faculty of Medicine, Universitas Sumatera Utara, Medan, Indonesia; e Intensive Care Unit Division, Imelda Pekerja Indonesia General Hospital, Medan, Indonesia

**Keywords:** Tuberculous ventriculitis, CNS tuberculosis, TB IRIS

## Abstract

**Introduction and importance:**

Tubercular Immune Reconstitution Inflammatory syndrome (TB-IRIS) is defined as the worsening of existing disease or new tuberculosis lesions during anti-tuberculosis therapy after excluding drug resistance, adherence issues, secondary infection, and malignancy. Ventriculitis is a rare and detrimental complication of cerebral tuberculosis. Here, we report a case of ventriculitis as a manifestation of TB-IRIS.

**Case presentation:**

A 46-year-old male presented to the emergency department with a decline in consciousness for four days prior to admission. He experienced a progressive headache accompanied by intermittent high-grade fever over the past week. He was diagnosed with rifampicin-sensitive pulmonary tuberculosis three months prior and was treated with a fixed-dose combination of anti-tuberculosis (ATT) regimen. His HIV test result was negative. A non-contrast computed tomography (CT) scan revealed ventriculitis and hydrocephalus. The patient subsequently received ATT and corticosteroids, along with external ventricular drainage (EVD) to alleviate intracranial pressure and address the intraventricular infection. Regrettably, the patient's condition progressively declined, resulting in his demise on the seventh day post-admission.

**Clinical discussion:**

TB-IRIS is primarily characterized in individuals with HIV/tuberculosis coinfection; however, it does not exclude that TB-IRIS may occur in immunocompetent conditions. Tuberculous ventriculitis is a manifestation of CNS TB-IRIS, characterized by significant morbidity and mortality. The fundamental principle in managing ventriculitis is to control both the inflammation and the infection and reducing intracranial pressure.

**Conclusion:**

This particular case does not significantly enhance the management of CNS-TB-IRIS; however, it does bring attention to the potential occurrence of this condition in immunocompetent patients.

## Highlights

• Tuberculous ventriculitis as a manifestation of CNS TB-IRIS in an immunocompetent patient
• The International Network for the Study of HIV-associated IRIS (INSHI) criteria may be used as a diagnostic tool.
• Corticosteroids, antitubercular therapy, and intracranial pressure are the primary considerations in management.

## 1 Introduction

Tuberculosis, the disease of the ancient, continues to be a significant global health issue due to its contagious nature, chronic progression, and complex immunologic reaction. [1] The standard medication used as first line therapy in managing tuberculosis is a four-drug regimen consisting of rifampicin, isoniazid, pyrazinamide, and ethambutol. This regimen has a cure rate of up to 95–98 % for drug-susceptible tuberculosis. [2] However, clinical deterioration may develop as an immunopathological response after a swift recuperation from anti-tuberculosis therapy (ATT). This condition, known as immune reconstitution inflammatory syndrome (IRIS), refers to the worsening of pre-existing tuberculosis lesions or the development of new tuberculosis lesions during the course of ATT, following the exclusion of factors such as drug resistance, adherence challenges, secondary infection, and malignancy. [3]. TB IRIS is generally associated with Human Immunodeficiency Virus (HIV) infection. The most accepted hypothesis is that TB treatment will increase the release of antigens by dying cells. If introduced at the same time, antiretroviral therapy will rapidly correct the immunosuppression related to HIV, resulting in an exaggerated immune response. In a person without HIV, the paradoxical inflammatory response is reported following withdrawal of immunosuppressant drugs such as corticosteroid and antitumor necrosis factor alpha therapy. However, it is not a common finding in immunocompetent patients. [4]

TB-IRIS can occur in any region of the body with a generally favorable outcome. Nevertheless, due to the complex nature of the CNS, a specific consideration is needed when managing CNS TB-IRIS. CNS TB IRIS can present as a wide range of pathology, including meningitis, tuberculoma, spinal epidural abscess, or radiculomyelitis, and is associated with a poor prognosis, which contributes to the majority of TB-IRIS-related mortality. [5,6] Tuberculous ventriculitis, also known as TB-ventricular empyema, is an infectious inflammatory condition affecting the ventricular system and possibly leading to hydrocephalus. It is a rare and under-recognized complication of CNS tuberculosis, with <10 reported cases in the English literature to date. Several authors proposed that tuberculous ventriculitis may arise from the rupture a tubercular abscess into the ventricular system or the existence of a subependymal tubercle. [7] Still, it possesses the potential to result in catastrophic consequences. [8] In this report, we present a case of tuberculous ventriculitis as a devastating manifestation of CNS-TB-IRIS in an immunocompetent patient. This work is reported according to the SCARE criteria and the revised 2020 SCARE guidelines. [9]

## 2 Case presentation

A 46-year-old man presented to the emergency unit with a decrease in consciousness. Initially, he complained of headaches that progressively worsened with intermittent high-grade fever around one to two weeks before. Furthermore, his consciousness had gradually decreased in the last four days. No seizure was reported. Over the previous three months, he was diagnosed with rifampicin-sensitive pulmonary tuberculosis based on GeneXpert MTB/RIF assay and treated with a 4-fixed drug combination (4-FDC, consisting of a daily dose of 600 mg of rifampicin, 300 mg of isoniazid, 1600 mg of pyrazinamide, and 1100 mg of ethambutol). He was part of a directly observed treatment short course (DOTS) program for ATT, and the medication adherence was good. Clinical improvement was noted, as cough was stopped during follow-up, and body weight was increased after TB medication.

**Fig. 1:**
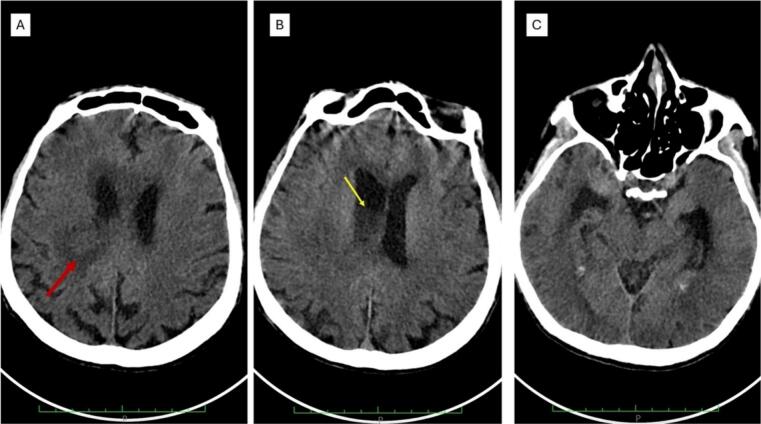
Noncontrast Head CT Scan. A fluid-fluid level was seen on the lateral ventricle (1A and B, arrow), consistent with ventriculitis. Dilatation of the lateral ventricle was also observed (C).

On physical examination, the patient was in a coma (GCS 7 out of 15) and had a fever (38,7C) with an increased heart rate (102 beats per minute). The neurological examination revealed nuchal rigidity with bilateral papilledema. The blood examination showed leukocytosis (WBC 10,200/mm3) and hyponatremia (Na^+^ serum 118 mEq/L), and the HIV test was negative. An emergency non-contrast CT scan of the brain showed a fluid-fluid level on the lateral ventricle, indicating the presence of pus in the lateral ventricle. The ventricular system was enlarged, indicating ventriculitis with hydrocephalus (Figs. 1A and B).

The patient was diagnosed with TB-IRIS based on the International Network for the Study of HIV-associated IRIS (INSHI) criteria, along with ventriculitis and hydrocephalus. We continued the ATT regimen and added high-dose prednisone (1.5 mg/kg/day) and administered a high-dose broad-spectrum antibiotic empirically. An external ventricular drainage (EVD) was placed on the right frontal to reduce the intracranial pressure and manage the infection. During ventriculostomy, the CSF was turbid and high pressure. Routine CSF examination revealed increased leukocyte (1397 mm3/μl; 93 % polymorphonuclear cells and 7 mononuclear cells), a slight increase of protein (0.2 g/dL), low glucose (15 mg/dL), and high lactate dehydrogenase (LDH) (836 U/L). Histopathologic examination revealed an inflammatory smear. Gram staining of the CSF showed no bacteria, and bacterial culture was negative. GeneXpert MTB/RIF assay of the CSF showed negative results. The patient was admitted to the intensive care unit for supportive treatment. However, the patient's condition gradually deteriorated, and he passed away on day seven after admission.

## 3 Discussion

The four-drug regimen, consisting of rifampicin, isoniazid, pyrazinamide, and ethambutol, has been widely adopted as the primary therapeutic approach for tuberculosis since its inception in the 1980s. The cure rate of drug-susceptible tuberculosis is significantly high, reaching up to 95–98 % under clinical trial conditions. [2] Nevertheless, an immune reconstitution inflammatory syndrome (IRIS) may arise during ATT treatment.

IRIS is defined as a worsening of the existing disease or the appearance of new tuberculosis lesions during anti-tuberculosis therapy after excluding drug resistance, adherence issues, secondary infection, and malignancy. [3] Generally, TB-IRIS is mainly described in patients with HIV/tuberculosis coinfection. However, there has been a growing number of reports regarding TB-IRIS in individuals with a fully functioning immune system, as evidenced in this specific case. Currently, there is no established guideline of TB-IRIS in HIV-negative patients. [10] The absence of a diagnostic test for IRIS necessitated the confirmation of diagnosis by utilizing case definition and clinical data. The diagnosis of IRIS was determined in this case due to the presence of new lesions during ATT, even though the initial symptoms of tuberculosis (cough and weight loss) had improved and there was good adherence to ATT. However, it is essential to note that drug susceptibility is vital in diagnosing IRIS. Clinically, it is challenging to distinguish TB-IRIS from drug resistance, as one report indicated that approximately 10 % of individuals diagnosed with TB-IRIS were a case of drug-resistant TB. [11] This issue is particularly significant given the high incidence of drug-resistant TB (around 18 %), especially in endemic regions. [12,13]

TB-IRIS can present itself as either of two distinct syndromes: (1) The occurrence of a paradoxical reaction after the initiation of antiretroviral therapy (ART) in individuals with tuberculosis (TB), or (2) the emergence of a novel tuberculosis manifestation known as “unmasking tuberculosis-associated IRIS” within a few weeks following the initiation of ART. As previously stated, tuberculosis paradoxical reactions can occur in both HIV and non-HIV patients, but with a significantly higher occurrence in HIV patients. [14] In HIV-uninfected individuals, the paradoxical response typically manifested 21–56 days after the initiation of ART, whereas in HIV-infected patients undergoing antiretroviral therapy, it took 2–4 week. [4] The International Network for the Study of HIV-associated IRIS (INSHI) has established a specific set of criteria to aid in the diagnosis of TB-IRIS, particularly in settings with limited resources. [15] A recent study has shown that the INSHI criteria have a reasonable accuracy in diagnosing TB-IRIS, with a sensitivity of 77 % and a specificity of 86 %. [16] In this study, the patient was diagnosed with TB-IRIS according to the (INSHI) criteria.

The exact underlying mechanism of TB-IRIS remains unidentified. TB-IRIS in HIV patients is believed to result from an exaggerated and uncontrolled inflammatory reaction to tuberculosis, which is triggered by the rapid recuperation of the immune system. Increased concentrations of pro-inflammatory cytokines and chemokines are crucial in guiding exaggerated inflammatory reactions in patients with TB-IRIS. [17] The precise mechanism in non-HIV patients remains incompletely comprehended. The confluence of host genetic susceptibility and immune-mediated alterations in the ability to react to tuberculin proteins, which are generated during rapid *Mycobacteria* demise, gives rise to an unmanageable inflammatory response, ultimately resulting in a paradoxical reaction. Predisposing factors encompass a diminished lymphocyte count upon the onset of ATT, advanced age, male sex, and the presence of anemia leading to the onset of immune reactivity in non-HIV individuals. [18]

The pathogenesis of CNS TB-IRIS may exhibit distinct characteristics due to the localized inflammatory response within the central nervous system (CNS). [19] CNS TB-IRIS commonly presents as meningitis, cerebral tuberculomas, or cerebral abscess, either newly developed or worsening and/or featuring raised intracranial pressure. Meningitis and tuberculoma are the most commonly observed conditions in cases of CNS TB-IRIS. In one study, tuberculoma was found in 52 % of cases, often alongside meningitis. [4,20] The interval between the onset of ATT and IRIS varied from 19 days to three months, with a longer interval observered among immunocompetent patients, as demonstrated in our scenario. [21]

In the present report, the patient exhibited tubercular ventriculitis and associated hydrocephalus. Tuberculous ventriculitis typically arises from the spread or rupture of a tubercle into the ventricular system. [22] Ventriculitis itself is linked to significant rates of mortality and morbidity. The most extensive study conducted thus far revealed an in-hospital mortality rate of up to 30 %, with neurological complications observed in around 60 % of the individuals who survived. [23] However, despite the fact that ventriculitis is recognized as a dreadful consequence of a variety of CNS infections, it has been largely overlooked thus far, as there are no agreed-upon diagnostic criteria. [24] Fever and meningeal sign, as well as CSF pleocytosis, as reported in this case, are among the most-accepted diagnostic criteria in ventriculitis. Magnetic resonance imaging (MRI) with gadolinium contrast is considered the most reliable method for diagnosing central nervous system (CNS) tuberculosis, including ventriculitis. [25] However, the access to this imaging technique is limited in Indonesia. [26] Nevertheless, the presence of pus was observed by CT Scan and later verified after the insertion of an EVD. Moreover, the diagnosis of TB-IRIS was established using both culture examination and GeneXpert testing.

However, we are aware of the limitations of the diagnosis of CNS TB itself. GeneXpert's sensitivity in diagnosing meningitis tuberculosis ranges from 61 to 85 %, [27] and it is generally accepted that negative CSF results do not rule out the diagnosis of tuberculosis itself. Furthermore, CSF culture only has a modest role in diagnosing meningitis tuberculosis, with sensitivity around 50–70 %. [28] So, diagnosing meningitis tuberculosis based on microbiological findings may be difficult. In addition to the criteria mentioned above, this patient also showed hyponatremia, which is prevalent in meningitis tuberculosis patients. [29,30]

The basic principle in managing ventriculitis is controlling the infection and inflammation and reducing the intracranial pressure. We applied an external ventricular drain (EVD) to regulate the intracranial pressure, as recommended in various studies. [31] On the contrary, the patient's condition failed to show any indications of improvement. Even in cases of tubercular hydrocephalus without ventriculitis, the evidence of a deep coma state (Glasgow Coma Scale score < 8) is associated with a negative prognosis. [32] The use of ventricular lavage, either using endoscopic or irrigation methods using the double-drain technique, is beneficial in treating pyogenic ventriculitis. [33] However, additional research is needed to determine their effectiveness in managing tubercular ventriculitis.

As previously indicated, neuroinflammation is a pivotal element in CNS TB, and it becomes even more intricate in the context of CNS TB-IRIS. [19,34] The major approach for managing TB-IRIS is high-dose corticosteroid therapy, which has been supported by controlled clinical trials. [35] Nonetheless, its effectiveness is restricted in certain instances, as found in this study. In this situation, using anti-TNF medications, such as infliximab, may have a positive effect; yet, it is essential to note that limited evidence is available to support this claim. [36] Thalidomide is the other alternative in managing the CNS inflammation in tuberculosis. However, its benefit is still unclear and the there is a significant risk of side effect, that warrants its regular use. [37,38]

## 4 Conclusion

This case underscored the complexities and severe consequences of CNS TB-IRIS in immunocompetent patient. Precise diagnosis and timely response are essential; nevertheless, the challenges in reliably detecting CNS TB infections highlight the necessity for enhanced diagnostic criteria and greater access to advanced imaging techniques. Furthermore, although corticosteroids are fundamental in the management of TB-IRIS, their efficacy is inconsistent, necessitating additional research into alternative treatments. Continued research and awareness are crucial for enhancing treatment strategies and improving outcomes for patients afflicted by these complex manifestations of tuberculosis.

## Author contribution

Andre Marolop Pangihutan Siahaan and Andika Pradana = Study concept, Data collection, and surgical management for the patient.

Bahagia Willibrordus Maria Nainggolan and David MR Silalahi = Writing- original draft preparation.

Marsal Risfandi and Ahmad Brata Rosa = Editing and writing.

Andre Marolop Pangihutan Siahaan = senior author and manuscript reviewer.

## Consent

The patient's relative provided written consent for publication. The editor-in-chief of this journal can review a copy of the consent on request.

## Ethical approval

Our institution waives ethical approval for case reports.

## Funding

There is no funding for this study.

## Declaration of competing interest

We declare no conflict of interest that could inappropriately influence this work.

## References

[1] Barberis I., Bragazzi N.L., Galluzzo L., Martini M. (2017). The history of tuberculosis: from the first historical records to the isolation of Koch’s bacillus. J. Prev. Med. Hyg..

[2] Verbeeck R.K., Günther G., Kibuule D., Hunter C., Rennie T.W. (2016). Optimizing treatment outcome of first-line anti-tuberculosis drugs: the role of therapeutic drug monitoring. Eur. J. Clin. Pharmacol..

[3] Guo T., Guo W., Song M., Ni S., Luo M., Chen P. (2019). Paradoxical reaction in the form of new pulmonary mass during anti-tuberculosis treatment: a case series and literature review. Infect. Drug Resist..

[4] Lanzafame M., Vento S. (2016). Tuberculosis-immune reconstitution inflammatory syndrome. J. Clin. Tuberc. Other Mycobact. Dis..

[5] Agarwal U., Kumar A., Behera D., French M.A., Price P. (2012). Tuberculosis associated immune reconstitution inflammatory syndrome in patients infected with HIV: meningitis a potentially life threatening manifestation. AIDS Res. Ther..

[6] Pepper D.J., Marais S., Maartens G., Rebe K., Morroni C., Rangaka M.X. (2009). Neurologic manifestations of paradoxical tuberculosis-associated immune reconstitution inflammatory syndrome: a case series. Clin. Infect. Dis..

[7] Obame F.L.O., Elmi S.M., Dokponou Y.C.H., Imbunhe N., El Attari S., Laaguili J. (2024). Primary tuberculous pyogenic ventriculitis in an immunocompetent patient: a case report. Surg. Neurol. Int..

[8] Vaziri S., Soleiman-Meigooni S., Rajabi J., Asgari A. (2016). Tuberculous ventriculitis: a rare complication of central nervous system tuberculosis. Int. J. Mycobacteriol..

[9] Sohrabi C., Mathew G., Maria N., Kerwan A., Franchi T., Agha R.A. (2023). The SCARE 2023 guideline: updating consensus surgical CAse REport (SCARE) guidelines. Int. J. Surg..

[10] Weber M.R., Fehr J.S., Kuhn F.P., Kaelin M.B. (2021). Approach for tuberculosis-associated immune reconstitution inflammatory syndrome in an HIV-negative patient. BMJ Case Rep..

[11] Meintjes G., Rangaka M.X., Maartens G., Rebe K., Morroni C., Pepper D.J. (2009). Novel relationship between tuberculosis immune reconstitution inflammatory syndrome and Antitubercular drug resistance. Clin. Infect. Dis..

[12] Naidoo K, Perumal R, Cox H, Mathema B, Loveday M, Ismail N, et al. The epidemiology, transmission, diagnosis, and management of drug-resistant tuberculosis—lessons from the South African experience. Lancet Infect. Dis. 2024;24:e559–75. doi:10.1016/S1473-3099(24)00144-0. 38527475

[13] Tiberi S., Utjesanovic N., Galvin J., Centis R., D’Ambrosio L., van den Boom M. (2022). Drug resistant TB – latest developments in epidemiology, diagnostics and management. Int. J. Infect. Dis..

[14] Meintjes G., Lawn S.D., Scano F., Maartens G., French M.A., Worodria W. (2008). Tuberculosis-associated immune reconstitution inflammatory syndrome: case definitions for use in resource-limited settings. Lancet Infect. Dis..

[15] Meintjes G., Lawn S.D., Scano F., Maartens G., French M.A., Worodria W. (2008). Tuberculosis-associated immune reconstitution inflammatory syndrome: case definitions for use in resource-limited settings. Lancet Infect. Dis..

[16] Stek C., Buyze J., Menten J., Schutz C., Thienemann F., Blumenthal L. (2021). Diagnostic accuracy of the INSHI consensus case definition for the diagnosis of paradoxical tuberculosis-IRIS. JAIDS J. Acquir. Immune Defic. Syndr..

[17] Quinn C.M., Poplin V., Kasibante J., Yuquimpo K., Gakuru J., Cresswell F.V. (2020). Tuberculosis IRIS: pathogenesis, presentation, and management across the Spectrum of disease. Life (Basel).

[18] Aggarwal D., Bhardwaj M., Kumar A., Saini V., Sawal N. (2020). Immune reconstitution inflammatory syndrome in non-HIV patients with tuberculosis. A case series. Indian J Tuberc.

[19] Marais S., Wilkinson K.A., Lesosky M., Coussens A.K., Deffur A., Pepper D.J. (2014). Neutrophil-associated central nervous system inflammation in tuberculous meningitis immune reconstitution inflammatory syndrome. Clin. Infect. Dis..

[20] Louncény Fatoumata B, Ibrahima Sory S, Fodé Abass C, Hamani Bachir DA, Ghislain AH, Alpha Youssouf C, et al. Cerebral abscesses of tuberculosis origin: study of 8 cases at the University Hospital of Conakry. Egypt. J. Neurosurg. 2020 35:1 2020;35:1–6. doi:10.1186/S41984-020-00090-X.

[21] van Toorn R., Rabie H., Dramowski A., Schoeman J.F. (2012). Neurological manifestations of TB-IRIS: a report of 4 children. Eur. J. Paediatr. Neurol..

[22] Marais S., Meintjes G., Pepper D.J., Dodd L.E., Schutz C., Ismail Z. (2013). Frequency, severity, and prediction of tuberculous meningitis immune reconstitution inflammatory syndrome. Clin. Infect. Dis..

[23] Luque-Paz D., Revest M., Eugène F., Boukthir S., Dejoies L., Tattevin P. (2021). Ventriculitis: a severe complication of central nervous system infections. Open Forum. Infect. Dis..

[24] Guanci M.M. (2013). Ventriculitis of the central nervous system. Crit. Care Nurs. Clin. North Am..

[25] Dahal P., Parajuli S. (2024). Magnetic resonance imaging findings in central nervous system tuberculosis: a pictorial review. Heliyon.

[26] Suthihono Y.A., Kusumastuti R.D. (2021). 2021 6th International Conference on Management in Emerging Markets (ICMEM).

[27] Hernandez A.V., de Laurentis L., Souza I., Pessanha M., Thota P., Roman Y.M. (2021). Diagnostic accuracy of Xpert MTB/RIF for tuberculous meningitis: systematic review and meta-analysis. Trop. Med. Int. Health.

[28] Ssebambulidde K., Gakuru J., Ellis J., Cresswell F.V., Bahr N.C. (2022). Improving technology to diagnose tuberculous meningitis: are we there yet?. Front. Neurol..

[29] Hieu T.H., Hashan M.R., Morsy S., Tawfik G.M., Cucè F., Sharma A. (2021). Hyponatremia in tuberculous meningitis: a systematic review and meta-analysis. Indian J. Tuberc..

[30] Misra U.K., Kalita J. (2021). Mechanism, spectrum, consequences and management of hyponatremia in tuberculous meningitis. Wellcome Open Res.

[31] Singh P., Paliwal V.K., Neyaz Z., Srivastava A.K., Verma R., Mohan S. (2014). Clinical and magnetic resonance imaging characteristics of tubercular ventriculitis: an under-recognized complication of tubercular meningitis. J. Neurol. Sci..

[32] Paliwal V., Garg R. (2021). Hydrocephalus in tuberculous meningitis - pearls and nuances. Neurol. India.

[33] Al Menabbawy A., El Refaee E., Soliman M.A.R., Elborady M.A., Katri M.A., Fleck S. (2020). Outcome improvement in cerebral ventriculitis after ventricular irrigation: a prospective controlled study. J. Neurosurg. Pediatr..

[34] Saghazadeh A., Rezaei N. (2022). Central inflammatory cytokines in tuberculous meningitis: a systematic review and meta-analysis. J. Interf. Cytokine Res..

[35] Meintjes G., Wilkinson R.J., Morroni C., Pepper D.J., Rebe K., Rangaka M.X. (2010). Randomized placebo-controlled trial of prednisone for paradoxical tuberculosis-associated immune reconstitution inflammatory syndrome. AIDS.

[36] Robert M., Mageau A., Gaudemer A., Thy M., Peiffer Smadja N., de Lastours V. (2024). Incidence, risk factors and treatment of central nervous system immune reconstitution inflammatory syndrome in <scp>non-HIV</scp> patients with tuberculous meningitis: a multicentre observational study. Intern. Med. J..

[37] Schoeman J.F., Springer P., van Rensburg A.J., Swanevelder S., Hanekom W.A., Haslett P.A.J. (2004). Adjunctive thalidomide therapy for childhood tuberculous meningitis: results of a randomized study. J. Child Neurol..

[38] van Toorn R., Solomons R.S., Seddon J.A., Schoeman J.F. (2021). Thalidomide use for complicated central nervous system tuberculosis in children: insights from an observational cohort. Clin. Infect. Dis..

